# Supplementary material for: Uricase-Expressing Engineered Macrophages Alleviate Murine Hyperuricemia
Source: Biomedicines. 2024 Nov 14;12(11):2602. doi: 10.3390/biomedicines12112602 (PMC11592275; doi:10.3390/biomedicines12112602)
Supplement: Supplementary file 1 [file biomedicines-12-02602-s001.zip › biomedicines-3276382-supplementary.pdf]

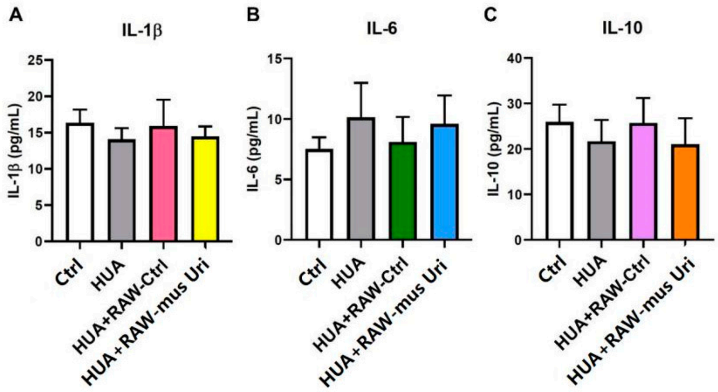

Supplementary Figure S1: Inflammatory factors between groups determined by ELISA. (**A**) IL-1β determined by ELSA showed no significant difference between the macrophage treated groups and the control groups. (**B**) IL-6 determined by ELSA showed no significant difference between the macrophage treated groups and the control groups. (**C**) IL-10 determined by ELSA showed no significant difference between the macrophage treated groups and the control groups.

Supplementary Table S1: UA levels in each group (μmol/L). A supplementary data to Figure 3.

	Ctrl	HUA	HUA + Ctrl	HUA + *mus*-Uri	HUA + Apo
0 day	119.8 ± 10.1	120.6 ± 18.5	124.0 ± 11.9	124.0 ± 12.9	126.3 ± 13.9
7 day	126.0 ± 10.1	193.2 ± 6.4	179.2 ± 5.0	117.4 ± 7.2	118.1 ± 6.3
14 day	138.0 ± 8.0	210.0 ± 24.4	185.4 ± 7.4	110.4 ± 16.1	109.2 ± 18.2
